# Prevalence and Characteristics of Extended-Spectrum β-Lactamase and Plasmid-Mediated Fluoroquinolone Resistance Genes in *Escherichia coli* Isolated from Chickens in Anhui Province, China

**DOI:** 10.1371/journal.pone.0104356

**Published:** 2014-08-20

**Authors:** Lin Li, Binting Wang, Shuai Feng, Jinnian Li, Congming Wu, Ying Wang, Xiangchun Ruan, Minghua Zeng

**Affiliations:** 1 Pharmacology and Toxicology Laboratory, College of Animal Science and Technology, Anhui Agricultural University, Hefei, P. R. China; 2 Microbiology and Immunology Laboratory, College of Animal Science and Technology, Anhui Agricultural University, Hefei, P. R. China; 3 Beijing Key Laboratory of Detection Technology for Animal-Derived Food Safety, College of Veterinary Medicine, China Agricultural University, Beijing, P. R. China; The University of Hong Kong, China

## Abstract

The aim of this study was to characterize the prevalence of extended-spectrum β-lactamase (ESBL) genes and plasmid-mediated fluoroquinolone resistance (PMQR) determinants in 202 *Escherichia coli* isolates from chickens in Anhui Province, China, and to determine whether ESBL and PMQR genes co-localized in the isolates. Antimicrobial susceptibility for 12 antimicrobials was determined by broth microdilution. Polymerase chain reactions (PCRs), DNA sequencing, and pulsed field gel electrophoresis (PFGE) were employed to characterize the molecular basis for β-lactam and fluoroquinolone resistance. High rates of antimicrobial resistance were observed, 147 out of the 202 (72.8%) isolates were resistant to at least 6 antimicrobial agents and 28 (13.9%) of the isolates were resistant to at least 10 antimicrobials. The prevalence of *bla*_CTX-M_, *bla*_TEM-1_ and *bla*_TEM-206_ genes was 19.8%, 24.3% and 11.9%, respectively. Seventy-five out of the 202 (37.1%) isolates possessed a plasmid-mediated quinolone resistance determinant in the form of *qnrS* (n = 21); this determinant occurred occasionally in combination with *aac(6′)-1b-cr* (n = 65). Coexistence of ESBL and/or PMQR genes was identified in 31 of the isolates. Two *E. coli* isolates carried *bla*_TEM-1_, *bla*_CTX-M_ and *qnrS*, while two others carried *bla*_CTX-M_, *qnrS* and *aac(6′)-1b-cr*. In addition, *bla*_TEM-1_, *qnrS* and *aac(6′)-1b-cr* were co-located in two other *E. coli* isolates. PFGE analysis showed that these isolates were not clonally related and were genetically diverse. To the best of our knowledge, this study is the first to describe detection of TEM-206-producing *E. coli* in farmed chickens, and the presence of *bla*_TEM-206_, *qnrS* and *aac(6′)-1b-cr* in one of the isolates.

## Introduction

The emergence of multidrug-resistant bacteria in the natural environment constitutes a serious risk to domestic animals and humans. Extended-spectrum β-lactamases (ESBLs) once constituted the most important cephalosporin resistance mechanism in *Enterobacteriaceae*, particularly *Escherichia coli* [1]. The majority of clinically isolated ESBLs are *bla*_TEM_, *bla*_SHV_ and *bla*_CTX-M_ types [2], [3], and CTX-M-producing *E. coli* isolates are recognized as the cause of hospital and community-onset infections [3].

Fluoroquinolones are broad-spectrum antimicrobial agents used for treating a variety of bacterial infections [4]. Three plasmid-mediated fluoroquinolone resistance (PMQR) mechanisms have been described; these include (i) the *Qnr* (*qnrA*, *qnrB*, *qnrS*, *qnrC* and *qnrD*) proteins, (ii) the *aac(6′)-Ib-cr* enzyme, and, (iii) *QepA* and *OqxAB* plasmid-mediated efflux pumps [5]. ESBL-producing isolates are commonly associated with PMQR genes in *Enterobacteriaceae* [6]. However, neither ESBL nor PMQR genes in *E. coli* isolates from chickens have been described in the Anhui Province. The main purpose of this study was to investigate the prevalence of ESBL and PMQR genes in *E. coli* collected recently from four chicken farms and characterization of the β-lactam and fluoroquinolone resistance mechanisms from isolates resistant to these drugs.

## Materials and Methods

### Bacterial isolates

In this study, *E. coli* isolates (n = 202) were collected from chicken cloacae at four different farms located in Anhui Province, China, from March 2012 to May 2012. The data and location for each farm are as follows: No. 1 chicken farm (n = 51, located in Hefei city), No. 2 chicken farm (n = 50, located in Changfeng county), No. 3 chicken farm (n = 50, located in Feixi county), and No. 4 chicken farm (n = 51, located in Feidong county). Sterile cotton swabs were used to collect fecal samples from chicken cloacae and the swabs were immediately transferred to sterile collection containers.

### Ethics statement

The study was carried out on private land (chicken farms), no specific permissions were required for these locations. The field studies did not involve endangered or protected species. The bacteria included in the study were obtained by routine procedures in each of the chicken farms involved. All procedures performed on the chickens were approved by China Agricultural University Animal Care and Use Committee.

### Antimicrobial susceptibility testing

The minimum inhibitory concentrations (MICs) of amoxicillin, ceftriaxome, ceftiofur, amikacin, gentamicin, apramycin, doxycycline, oxytetracycline, florfenicol, enrofloxacin, ofloxacin and lomefloxacin were determined using the broth microdilution method according to the guidelines issued by the Clinical and Laboratory Standards Institute [7]. *E. coli* ATCC 25922 was used as a quality control strain.

### Resistance genotype characterization

ESBL genes (*bla*_CTX-M_, *bla*_TEM_ and *bla*_SHV_) and PMQR genes (*qnrA*, *qnrB*, *qnrC*, *qnrD*, *qnrS*, *aac(6′)-Ib-cr* and *qepA*) were detected by polymerase chain reaction (PCR) with the gene-specific primers listed in Table 1. All of the PCR amplicons were confirmed by dideoxy DNA sequencing. The DNA sequences obtained were compared with those in GenBank using the BLAST program (http://blast.ncbi.nlm.nih.gov/).

**Table 1:** PCR Primers and annealing temperatures.

*Gene*	*Sequence (5′-3′)*	*Annealing temperature (°C)*	*Reference*
*Bla*_TEM_	F: ATGAGTATTCAACATTTCCG R: CCAATGCTTAATCAGTGAGG	50	
*bla*_CTX-M_	F: CGATGGGACGATGTCACTG R: CGGCTTTCTGCCTTAGGTT	59	
*Bla*_SHV_	F: TATCTCCCTGTTAGCCACCC R: CGCCTCATTCAGTTCCGTTT	55	
*qnrA*	F: AGAGGATTTCTCACGCCAGG R: TGCCAGGCACAGATCTTGAC	54	[8]
*qnrB*	F: GGAATCGAAATTCGCCACTG R: TTTGCCGTTCGCCAGTCGAA	58	
*qnrC*	F: GGTTGTACATTTATTGAATC R: TCCACTTTACGAGGTTCT	50	[9]
*qnrD*	F: AGATCAATTTACGGGGAATA R: AACAAGCTGAAGCGCCTG	50	[10]
*qnrS*	F: CACTTTGATGTCGCAGAT R: CAACAATACCCAGTGCTT	52	[11]
*aac(6′)-Ib-cr*	F: GATGCTCTATGGGTGGCTAA R: GGTCCGTTTGGATCTTGGTGA	58	
*qepA*	F: CCGATGACGAAGCACAGGG R: CTACGGGCTCAAGCAGTTGG	50	

### Pulsed-field gel electrophoresis (PFGE) analysis

Genomic DNA from each isolate was analyzed by PFGE after digestion with the restriction enzyme *Xba*I (TaKaRa Dalian, Liaoning, China). Electrophoresis conditions comprised 6.0 V/cm with an initial/final switch time of 2.16 sec/54.17 sec and an angle of 12 at 14°C for 18.5 h. *Salmonella enterica* serovar Braenderup H9812 standards served as size markers. PFGE patterns were analyzed using the PulseNet Standardized Laboratory Protocol and the CHEF MAPPERTM System (Bio-Rad Laboratories, Hercules, CA). Dice similarity coefficients were calculated and the unweighted pair group method with arithmetic averages (UPGMA) was used for cluster analysis. 1.5% optimization and 1.0% position tolerance were used.

## Results

### Antimicrobial susceptibility of *E. coli* isolates

**Figure 1:**
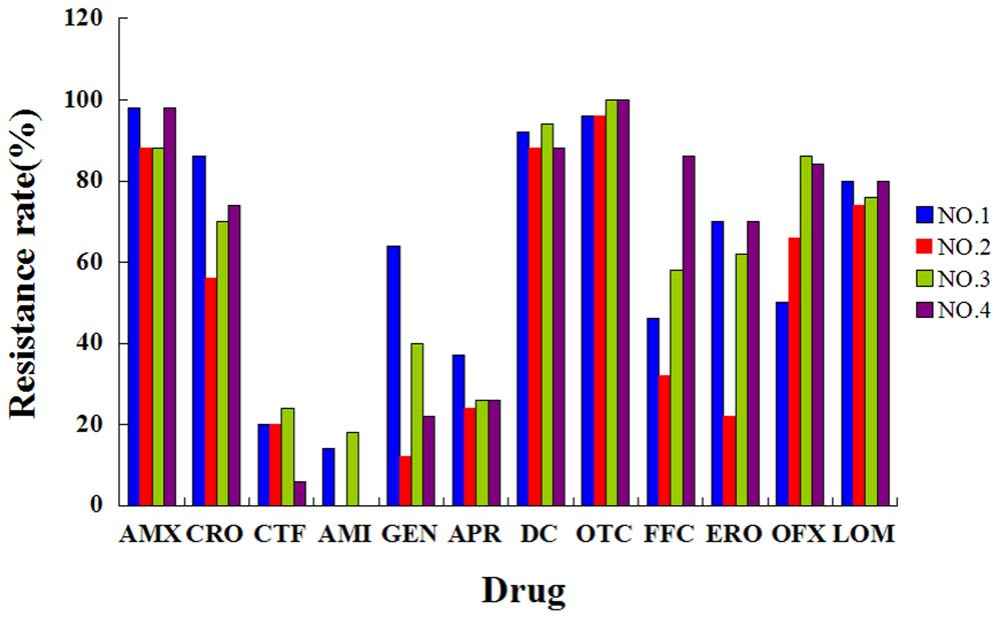
Resistance rates of *Escherichia coli* isolates from four chicken farms to 12 antimicrobials. Abbreviations: AMX: amoxicillin, CRO: ceftriaxome, CTF: ceftiofur, AMI: amikacin, GEN: gentamicin, APR: apramycin, DC: doxycycline, OTC: oxytetracycline, FFC: florfenicol, ERO: enrofloxacin, OFX: ofloxacin, LOM: lomefloxacin.

**Figure 2:**
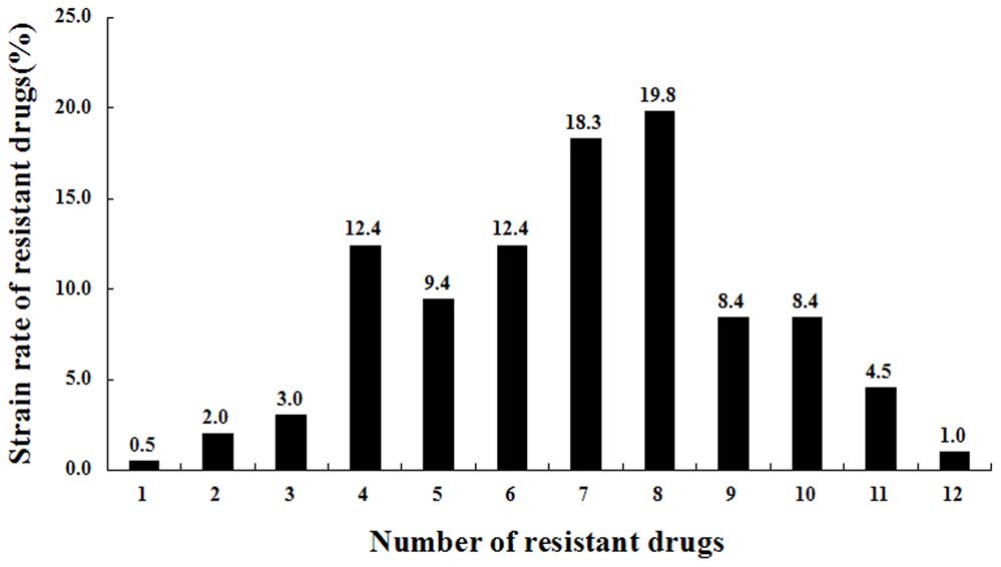
Resistance profiles of 202 *Escherichia coli* isolates from four chicken farms to 12 antimicrobials. Note: one to twelve in the X axis represents resistance to one antimicrobial to twelve antimicrobials. Y axis represents the rates of the isolates resistance to one antimicrobial to twelve antimicrobials.

High rates of resistance to oxytetracycline (98.0%), amoxicillin (93.5%), doxycycline (90.6%), lomefloxacin (77.5%), ceftriaxome (70.3%), ofloxacin (68.8%), enrofloxacin (56.4%), and florfenicol (53.5%) were observed in the 202 *E. coli* isolates. Low rates of resistance to gentamicin (34.5%), apramycin (28.0%), ceftiofur (17.5%) and amikacin (8.4%) were observed. Resistance rates of *Escherichia coli* isolates from four chicken farms to 12 antimicrobials can be seen in Fig. 1. One hundred and forty-seven (72.8%) of the isolates were resistant to at least 6 antimicrobial agents (72.8% = 12.4+18.3+19.8+8.4+8.4+4.5+1.0%), while 28 (13.9%) were resistant to at least 10 of these drugs (13.9% = 8.4+4.5+1.0%) (Fig. 2).

### Characterization of ESBL and PMQR determinants

β-lactamase encoding genes were detected in 100 of the 202 (49.5%) isolates (*bla*_CTX-M_ (n = 40), *bla*_TEM-1_ (n = 49) and *bla*_TEM-206_ (n = 24), among which *bla*_CTX-M_ and *bla*_TEM-1_ were co-located in 13 isolates), but no isolates were positive for the *bla*_SHV_ gene. Seventy-five out of the 202 (37.1%) isolates possessed plasmid-mediated quinolone resistance determinants. The positive rates for *qnr* genes and *aac(6′)-Ib-cr* were 10.4% (n = 21) and 32.2% (n = 65), respectively. Among the *qnr* determinants, only the *qnrS*-type gene was detected; no isolates were positive for *qnrA*, *qnrB*, *qnrC*, *qnrD*, and *qepA* genes.

### Coexistence of ESBL and/or PMQR genes and genetic relationships in the isolates

Coexistence of ESBL and/or PMQR genes was identified in 31 *E. coli* isolates (Table 2), which were mainly from chicken farm No 3. Coexistence of *bla*_TEM-1_, *bla*_CTX-M_ and *qnrS* was observed in two *E. coli* isolates exclusively from the same chicken farm. Hence, coexistence of *bla*_CTX-M_, *qnrS* and *aac(6′)-1b-cr* and coexistence of *bla*_TEM-1_, *qnrS* and *aac(6′)-1b-cr* was observed. In addition, coexistence of *bla*_TEM-206_, *qnrS* and *aac(6′)-1b-cr* was observed in one *E. coli* isolate from chicken farm No. 3.

**Table 2:** Coexistence of ESBL and/or PMQR genes.

*Coexistence of ESBL and/or PMQR genes*	*No. of strains carrying co-localized resistance genes (%)*	*chicken farm designation*
*bla*_TEM_ *bla*_CTX-M_	11 (5.4)	NO. 1, NO. 3
*bla*_TEM_ *qnrS*	2 (1.0)	NO. 3
*bla*_TEM_ *aac(6′)-Ib-cr*	2 (1.0)	NO. 3
*bla*_CTX-M_ *aac(6′)-Ib-cr*	3 (1.5)	NO. 2, NO. 3, NO. 4
*qnrS aac(6′)-Ib-cr*	6 (3.0)	NO. 3, NO. 4
*bla*_TEM-1_ *bla*_CTX-M_ *qnrS*	2 (1.0)	NO. 3
*bla_TEM-1_ qnrS aac(6′)-Ib-cr*	2 (1.0)	NO. 3
*bla_TEM-206_ qnrS aac(6′)-Ib-cr*	1 (0.5)	NO. 3
*bla*_CTX-M_ *qnrS aac(6′)-Ib-cr*	2 (1.0)	NO. 3

### PFGE profiles

**Figure 3:**
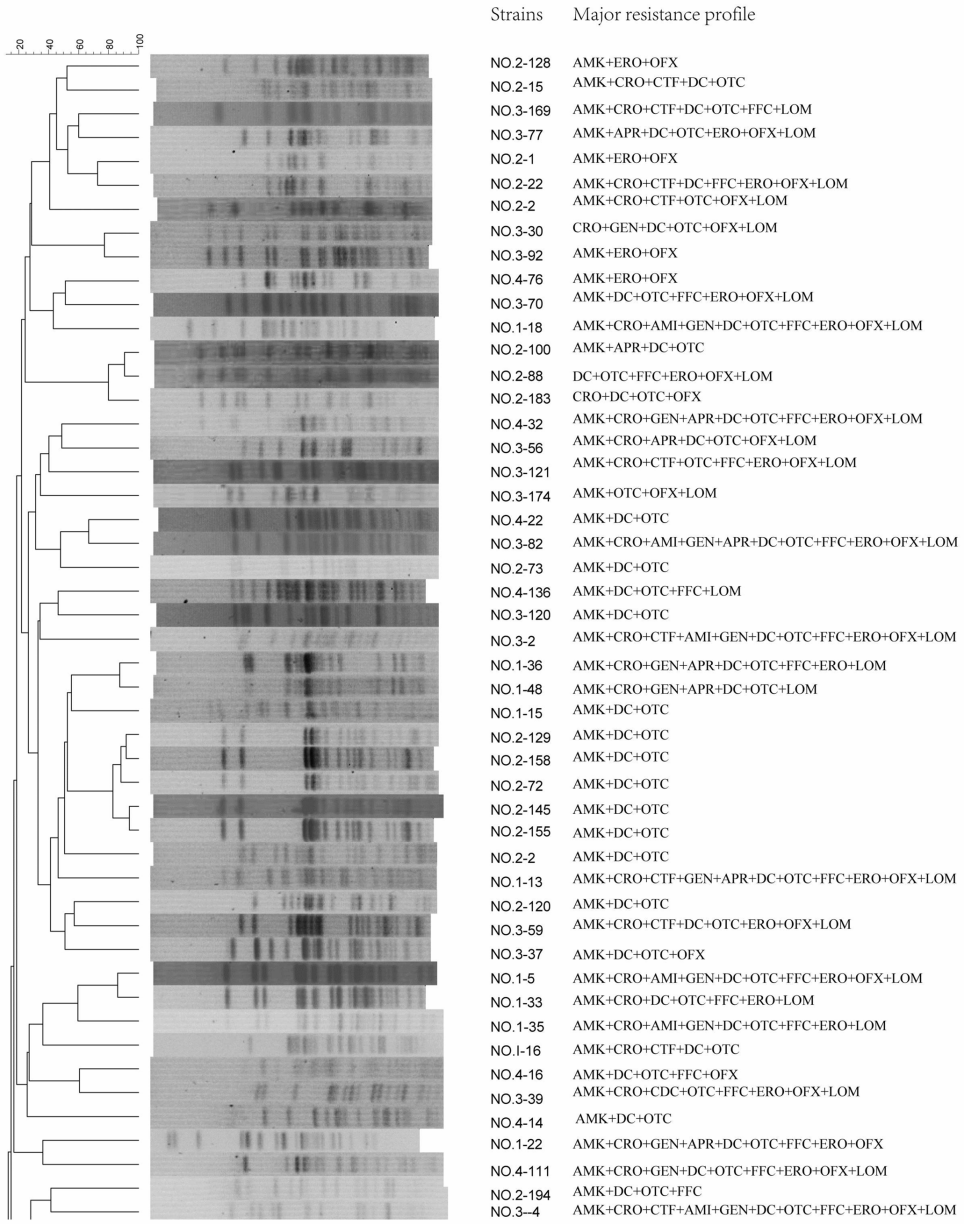
Comparison of the *Xba*I-PFGE patterns of 49 *Escherichia coli* isolates from chicken feces from chicken farms in the Anhui Province of China.

ESBL- and PMQR-positive isolates showed remarkable genomic diversity as revealed by PFGE (Fig. 3). The 49 ESBL- or PMQR-positive isolates exhibited 43 major patterns: eight patterns were observed in ten isolates from chicken farm No. 1, 13 patterns were observed in the 17 isolates from chicken farm No. 2, 15 patterns were observed in the 15 isolates from chicken farm No. 3, while 7 patterns were observed in the 7 isolates from chicken farm No. 4, indicating that most of these isolates were not clonally related. Some of the ESBL- or PMQR-positive isolates could not be PFGE typed; this may have been caused by DNA degradation in the samples.

## Discussion

Chicken is one of the most important food-producing animals. The inappropriate use and abuse of antibiotics in poultry husbandry lead to enhanced antibiotic resistance. ESBL and PMQR genes in *E. coli* from chickens had emerged and spread rapidly worldwide and posed a threat to human health through the food chain. However, there are no published data on the incidence of resistance to different antibiotics or the prevalence of ESBL and PMQR genes in *E. coli* from chickens in Anhui Province. Hence, farmed chickens were selected for epidemiological sampling to investigate antibiotic resistance in this region. In this study, 202 *E. coli* isolates were collected from chickens for genotyping. One hundred and forty-seven (72.8%) of the isolates were resistant to at least 6 antimicrobial agents, while 28 (13.9%) were resistant to at least 10 antimicrobials, indicating a high prevalence of high-level resistant *E. coli* species in chickens in Anhui Province. The high prevalence of ESBL and PMQR genes in recent years could be caused by overuse of various antimicrobials in chickens, a finding that is consistent with a report from China on monkeys in zoos by Wang et al. [8]. A study by Dai *et al.* [9] showed that the resistance rates in chickens for doxycycline, enrofloxacin, and amikacin were 75.0%, 67.5%, and 6.5%, respectively, between 2001 and 2006 in the Shandong Province of China. Additionally, Xia *et al.* reported enrofloxacin, amikacin, and gentamicin resistance rates of 99.0%, 27.8%, and 97%, respectively [10]. As noted in Henan Province, antibiotic resistance in chickens towards enrofloxacin, amikacin, and gentamicin were 80.6%, 80.6% and 87.1%, respectively [11]. In the present study, the following levels of resistance were observed: doxycycline (90.6%), enrofloxacin (70.3%), amikacin (8.4%), enrofloxacin (56.4%) and gentamicin (34.5%). Hence, the levels of resistance measured herein differed from surveys performed between 2001 and 2007 in China, especially for amikacin and gentamicin. The reasons for this variation could be related to differences in the geographical locations, farm environments and antimicrobial usage in these chicken farms.

ESBL genes did not emerge in *E. coli* of farm animal origin in China until 2004 [12]. Among the ESBL determinants, *bla*_CTX-M_ genes were detected in 40 (19.8%) of the isolates and these were the most prevalent ESBLs in this study, while CTX-M-producing *E. coli* isolates among farm animals in China was detected in 14 (2.4%) strains in 2007 [13]. This is consistent with the dissemination of this type of ESBL gene in European and Asia countries [14]–[17]. 55.6% (15) of the isolates resistant to more than 9 antimicrobial agents carried *bla*_CTX-M_ in the present study. This supported the argument that the acquisition of multi-drug-resistant plasmids plays a pivotal role in the dissemination of CTX-M ESBLs [18]. To our knowledge, this is the first study to describe detection of TEM-206-producing *E. coli* isolated from chickens. *Bla*_TEM-206_ was detected in 24 (11.9%) strains and *bla*_TEM-1_ was detected in 49 (24.3%) strains. This indicated that *bla*_TEM-1_ is the most common β-lactamase gene among *E. coli* isolates in China. This was in agreement with previous findings [8]. Hence, *bla*_TEM_ and *bla*_CTX-M_ type ESBL genes were the most common genotypes in this study.

With respect to PMQR genes, Seventy-five out of the 202 (37.1%) isolates possessed them, *qnrS* and *aac(6′)-Ib-cr* were dominant while other PMQR genes were not detected in this study. A study by Tong *et al.* showed that the prevalence of PMQR among isolates from chickens were 42.4% during 2004 to 2011 in China [19]. However, PMQR genes were detected in 5 (5.3%) of 94 *E. coli* chicken isolates in Turkey [20], and in 15 (15.6%)of 96 *E. coli* in 2006 in Nigeria [21]. Plasmid mediated quinolone resistances (*qnrS*) were found in 2(1.3%) of 151 *E. coli* chicken strains in Slovakia [22]. The frequency of PMQR genotypes in our study(37.1%) is lower than the report in China [19] and higher than that in Turkey,Nigeria and Slovakia. Typically, *qnrB* was considered to be the most prevalent PMQR gene in *Enterobacteriaceae* isolates in 2009 [5]. In the present study, the *aac(6′)-Ib-cr* enzyme was the most prevalent plasmid-mediated mechanism of quinolone resistance, as has been noted elsewhere [10]. The prevalence of this gene is higher than that reported by older surveys [10], [23]–[25]. Because of the high prevalence of PMQR genes and the relatively high levels of resistance to fluoroquinolones, it is important to intensify fluoroquinolone surveillance.

Notably, two *E. coli* isolates carried *bla*_TEM-1_, *bla*_CTX-M_ and *qnrS*, while two others carried *bla*_CTX-M_, *qnrS* and *aac(6′)-1b-cr*. In addition, *bla*_TEM-1_, *qnrS* and *aac(6′)-1b-cr* were co-located in two other *E. coli* isolates. To date, this is the first time that *bla*_TEM-206_, *qnrS* and *aac(6′)-1b-cr* have been found to co-localize in an individual *E. coli* isolate. All of the seven *E. coli* isolates came from chicken farm No. 3 where environmental hygiene was the poorest among the four farms. As is known to all, the concentration of ammonia gas is a distinguishable biological parameter to assess the hygiene condition in chicken house. The ammonia concentration (24 ppm) in chicken house in farm No. 3 is higher than that of the other three chicken farms which were 10 ppm, 13 ppm and 15 ppm, respectively (p<0.05). Chickens in farm No. 3 were free-ranging in their environments, where chicken faeces contamination make it easy for resistance genes and/or resistance intestinal bacteria to spread between chickens, while in the other three chicken farms the intensive feeding was managed and everyday clearance of the faeces helps to minimize the risk of infection. Therefore, there was a relatively higher death rate (13%) in farm No. 3 compared with that of the other three chicken farms which death rate were 6%, 9% and 10%, respectively (p<0.05). Hence it appears that the poor farm environment promoted the transfer of these resistance elements. In all, 31 *E. coli* isolates carried more than one gene encoding ESBLs and/or PMQR.

One hundred and forty-seven out of the 202 (72.8%) isolates analyzed herein were resistant to at least six antimicrobial agents while the results of the PFGE showed that the ESBL- and/or PMQR-positive isolates exhibited genomic diversity. The drug usage records of the four farms from which the *E.coli* isolates originated were in agreement with the resistance patterns of these strains. In these four farms, β-lactams, tetracyclines and fluoroquinolones had been commonly used for curing the chickens. During these drugs, amoxicillin, ceftriaxome, oxytetracycline, doxycycline, lomefloxacin, ofloxacin and enrofloxacin were more frequently consumpted and high rates of resistance to these drugs were observed and the selective pressure imposed by these antimicrobial agents might be the driving force for the prevalence of the resistance genes detected in these farms. However, low rates of resistance to aminoglycoside antibiotics including apramycin and amikacin were observed in these four farms, maybe due to the lesser use of these drugs. Resistance to 12 antimicrobials of isolates from the four chicken farms has no significant difference. As the PFGE profiles did not show any major clusters among the four chicken farms, the clonal transfer of resistant strains was not present in these four farms. As whether the resistance genes could be transmitted horizontally in vehicles such as plasmids, further studies should be conducted.

To the best of our knowledge, this is the first report where both ESBL and PMQR genes were located in *E. coli* isolated from chickens in Anhui Province. In addition, this is the first study to describe detection of TEM-206-producing *E. coli* isolated from chickens and where *bla*_TEM-206_, *qnrS* and *aac(6′)-1b-cr* were co-located in one *E. coli* isolate. In summary, widespread detection of ESBL and PMQR determinants and a high prevalence of antimicrobial resistance were evident in *E. coli* strains from chickens in Anhui Province, China.
